# Multimorbidity clusters among people with serious mental illness: a representative primary and secondary data linkage cohort study

**DOI:** 10.1017/S003329172200109X

**Published:** 2023-07

**Authors:** Ruimin Ma, Eugenia Romano, Mark Ashworth, Mohammad E. Yadegarfar, Alexandru Dregan, Amy Ronaldson, Claire de Oliveira, Rowena Jacobs, Robert Stewart, Brendon Stubbs

**Affiliations:** 1 Department of Psychological Medicine, Institute of Psychiatry, Psychology and Neuroscience (IoPPN), King's College London, London, UK; 2 South London and Maudsley NHS Foundation Trust, Denmark Hill, London, UK; 3 School of Life Course and Population Sciences, Faculty of Life Sciences and Medicine, King's College London, London, UK; 4 Health Services and Population Research Department, Psychology and Neuroscience (IoPPN), King's College London, London, UK; 5 Centre for Health Economics, University of York, York, UK; 6 Physiotherapy Department, South London and Maudsley National Health Services Foundation Trust, London, SE5 8AB, UK

**Keywords:** Mortality, multimorbidity, physical health, psychosis, schizophrenia

## Abstract

**Background:**

People with serious mental illness (SMI) experience higher mortality partially attributable to higher long-term condition (LTC) prevalence. However, little is known about multiple LTCs (MLTCs) clustering in this population.

**Methods:**

People from South London with SMI and two or more existing LTCs aged 18+ at diagnosis were included using linked primary and mental healthcare records, 2012–2020. Latent class analysis (LCA) determined MLTC classes and multinominal logistic regression examined associations between demographic/clinical characteristics and latent class membership.

**Results:**

The sample included 1924 patients (mean (s.d.) age 48.2 (17.3) years). Five latent classes were identified: ‘substance related’ (24.9%), ‘atopic’ (24.2%), ‘pure affective’ (30.4%), ‘cardiovascular’ (14.1%), and ‘complex multimorbidity’ (6.4%). Patients had on average 7–9 LTCs in each cluster. Males were at increased odds of MLTCs in all four clusters, compared to the ‘pure affective’. Compared to the largest cluster (‘pure affective’), the ‘substance related’ and the ‘atopic’ clusters were younger [odds ratios (OR) per year increase 0.99 (95% CI 0.98–1.00) and 0.96 (0.95–0.97) respectively], and the ‘cardiovascular’ and ‘complex multimorbidity’ clusters were older (ORs 1.09 (1.07–1.10) and 1.16 (1.14–1.18) respectively). The ‘substance related’ cluster was more likely to be White, the ‘cardiovascular’ cluster more likely to be Black (compared to White; OR 1.75, 95% CI 1.10–2.79), and both more likely to have schizophrenia, compared to other clusters.

**Conclusion:**

The current study identified five latent class MLTC clusters among patients with SMI. An integrated care model for treating MLTCs in this population is recommended to improve multimorbidity care.

## Introduction

People with serious mental illness (SMI) experience higher premature mortality, 10–20 years of life lost compared to the general population (Firth et al., 2019; Onyeka, Collier Høegh, Nåheim Eien, Nwaru, & Melle, 2019; Scott & Happell, 2011; Walker, McGee, & Druss, 2015). One of the key reasons accounting for it is the increased physical health burden in this population (Firth et al., 2019; Lawrence, Hancock, & Kisely, 2013). In fact, research has demonstrated an increased prevalence of long-term conditions (LTCs) in people with SMI (Correll et al., 2017; Firth et al., 2019; Onyeka et al., 2019; Scott & Happell, 2011; Suetani et al., 2021; Vancampfort et al., 2016, 2015).

The reasons for the poorer physical health are complex (Firth et al., 2019), ranging from socioeconomic conditions (Public Health England, 2018) to diagnostic overshadowing, health behaviours, and patients management (Firth et al., 2019; Solmi et al., 2021; Woodhead, Ashworth, Schofield, & Henderson, 2014). Despite the increased risk of LTCs and their impact on mortality, people with SMI are often screened less or have reduced access to screenings (Solmi et al., 2021, 2020), and fragmentated physical healthcare (Bradford et al., 2008; Firth et al., 2019).

Despite a five-fold increased prevalence of having three or more individual LTCs in people with SMI compared to the general population (Public Health England, 2018), relatively little research has considered the prevalence, clustering and impact of multiple LTCs (MLTCs) or multimorbidity in people with SMI. Multimorbidity, which is the co-occurrence of MLTCs (Foguet-Boreu et al., 2014; Van Den Akker, Buntinx, & Knottnerus, 1996), is associated with several deleterious consequences, including functional decline (Ryan, Wallace, O'Hara, & Smith, 2015), poor quality of life (Fortin et al., 2004), premature mortality and increased healthcare utilisation and costs (Lehnert et al., 2011; Soley-Bori et al., 2021). Multimorbidity further complicates the management of physical healthcare across multiple specialties and may further increase the mortality gap in those with SMI (Stubbs et al., 2016). The development of multimorbidity in the general population is influenced by a multitude of factors (Melaku et al., 2016; Salisbury, Johnson, Purdy, Valderas, & Montgomery, 2011; Vancampfort et al., 2017). Research in the general population has identified that multimorbidity can appear as different clusters of MLTCs such as clusters of cardiovascular and metabolic diseases, or musculoskeletal disorders (Prados-Torres, Calderón-Larrañaga, Hancco-Saavedra, Poblador-Plou, & Van Den Akker, 2014). Clusters of MLTCS have been shown to be associated with poorer quality of life (Sprangers et al., 2000), as well as patient burden, medication adherence and inability to work (Hajat & Stein, 2018).

Literature reports how people with SMI have substantially poorer physical health and a high burden of individual LTCs (de Oliveira, Mason, & Kurdyak, 2020; Firth et al., 2019), so understanding the patterns and clustering of MLTCs is important for treatment and prevention strategies in people with SMI. However, there is minimal evidence on the association of SMI with MTLCs/multimorbidity (Stubbs et al., 2017, 2016), and the occurring patterns of LTCs in this population. One study examined patterns of co-/multimorbidity in individuals with SMI compared with controls, reporting that although those with SMI were more likely to have individual physical conditions, but the overall physical condition patterns were identical between the two groups (Woodhead et al., 2014). A large cross-sectional multinational study found that people with psychosis were more likely to have multimorbidity and at a much younger age than the general population (Stubbs et al., 2016). A study of specialist mental health records found that the clusters of MLTCs in psychosis most strongly associated with mortality were cardiovascular-respiratory, neurologic-respiratory and respiratory-skin clusters (Kugathasan et al., 2020). Finally, a Danish nationwide study identified respiratory, digestive, and cardiovascular diseases as those most strongly associated with mortality in a sample of patients with schizophrenia (Kugathasan et al., 2019).

Despite the aforementioned, no study to date has examined the patterns and clusters of complex multimorbidity (i.e. with SMI and at least two co-existing LTCs). Studies to date have rarely considered mental health comorbidities while examining clusters of multimorbidity in populations with SMI, although both depression and anxiety are commonly identified as comorbidities in this population (Firth et al., 2019). There is also a lack of information regarding factors that may affect the development of clusters. This impedes the international efforts to improve the identification, management, and integration of physical healthcare in SMI and specifically address the premature mortality gap. Given this, we undertook a latent class analysis (LCA) in representative electronic health records to elucidate the clusters of LTCs in people with SMI, examining those patients with at least two additional LTCs recorded on top of their SMI, and thus focusing on more complex LTCs clusters. In this paper, multimorbidity patterns were explored by listing and ranking individual health conditions according to the proportion of SMI patients clinically diagnosed with each condition.

## Methods

### Data sources

Data for this observational cohort study were retrieved from linked electronic health records (EHRs) from primary and secondary mental health care drawn from services covering a defined geographic catchment area. The primary care data were obtained from Lambeth DataNet (LDN), a database containing 96.8% of primary care data in the borough of Lambeth, south London, and including more than 827 000 registered adult patients. The remaining 3.2% of patients had ‘informed dissent’ codes in their primary care record, preventing anonymised data extraction and analysis for research purposes. LDN provides pseudonymised clinical data including socio-demographic information (e.g. age, ethnicity, gender and deprivation level), consultations, service referrals and medications (Dorrington et al., 2020). The borough of Lambeth is the 9^th^ (out of 32 boroughs) and 44^th^ (out of 309 boroughs) most deprived borough in London and England, respectively (Lambeth Council, 2017), and comprises an ethnically diverse population, with a large proportion of mixed ethnic (7.6%) and black communities (25.9%) (Census Information Scheme, 2015; Woodhead et al., 2014).

The primary care data from LDN have been linked to EHR data from specialist mental health care from the South London and Maudsley NHS Foundation Trust (SLaM) Clinical Record Interactive Search (CRIS) (Perera et al., 2016). SLaM is one of the largest mental health care providers in Europe, serving a geographic catchment of four boroughs in South London, including the entirety of the borough of Lambeth covered by LDN. De-identified EHR-derived data in CRIS represent over 500 000 people who have received care from SLaM, which provides comprehensive publicly funded services to its catchment. Data from structured fields in the source EHR have been extensively supplemented by natural language process (NLP) applications using Generalised Architecture for Text Engineering software, applying information extraction techniques to the extensive text fields held in the clinical notes (Perera et al., 2016, p. 20). CRIS has full approval for secondary analysis (Oxford Research Ethnic Committee C, reference 18/SC/0372), and the SLaM Biomedical Research Centre (BRC) Case Register has been described in detail elsewhere (Perera et al., 2016; Stewart et al., 2009). The linkage between LDN and CRIS is regularly updated and stored by the SLaM Clinical Data Linkage Service (CDLS), a local trusted safe haven service (Woodhead et al., 2016).

### Study cohort

People with SMI in CRIS were identified on the basis of International Classification of Disease, Tenth Revision (ICD-10) diagnoses of bipolar affective disorder, mania, schizophrenia, schizoaffective disorder, and other psychotic disorders (ICD-10 codes F20*-25* and F28*-31; World Health Organization, 2009). SMI in LDN was determined by using relevant Read codes for SMI (UK Government, 2015). Eligible patients included those aged 18 and over at the time of first SMI diagnosis and who had a record in both CRIS and LDN between the period of 1^st^ April 2012 and 1^st^ March 2020. To avoid the impact of COVID-19 on the screening and presentation of LTCs in the UK, pre-COVID-19 study period was decided. Additionally, patients were required to have at least two co-existing LTCs (as defined below) on top of their SMI diagnosis in LDN within the study period. Individuals enter the cohort at the latest date of the onset of either SMI or their second LTC, whichever order they occur in, was served as index date.

### Measures

#### Long-term conditions (LTCs)

Within the group with SMI, data were extracted on 35 LTCs proposed and characterised in previous research (Cassell et al., 2018). The 35 LTCs were extracted from LDN and defined by Read codes and dm + d codes (i.e. a dictionary of codes and descriptions for devices and medication used in NHS Primary Care [MacKenna, 2019]; chronic pain was defined by dm + d codes only). The list of LTCs included in our study were: HIV and/or AIDS, alcohol problems, anorexia and/or bulimia, asthma, atrial fibrillation, blindness and low vision, bronchiectasis, cancer, chronic obstructive pulmonary disease (COPD), chronic pain, chronic sinusitis, coronary heart disease, dementia, depression and/or anxiety, diabetes, diverticular disease of intestine, epilepsy, hearing loss, heart failure, hypertension, inflammatory bowel disease (IBD), irritable bowel syndrome (IBS), kidney disease, learning disabilities, liver disease and hepatitis, multiple sclerosis (MS), other inflammatory polyarthropathies and systematic connected tissue disorders, peptic ulcer, Parkinson's disease, peripheral vascular disease, psoriasis and/or eczema, psychoactive substance dependency, rheumatoid arthritis, stroke, and thyroid disorders.

#### Covariates

Socioeconomic and demographic covariates were extracted from CRIS, including age at index date, gender, and ethnicity. Age was calculated for each case based on date of birth and date of data extraction. Ethnicity was categorised into four categories (White, Black, Asian, Mixed/other). The 2011-defined lower super output area (LSOA) was extracted from LDN. These are geographic areas of residence covering an average of 1722 people (Greater London Authority, 2014), implemented to improve the reporting of small area statistics in England and Wales (Office of National Statistics, 2015). LSOAs were extracted to assign a standard deprivation score, the Index of Multiple Deprivation (IMD-2019, derived at this geographic unit from individual Census data), for each case based on his/her area of residence closest to the time of initial SMI diagnosis between the period of 1^st^ April 2012 and 1^st^ March 2020 (either prior to or after diagnosis).

### Statistical analysis

LCA was conducted to determine different multimorbidity classes (Weller, Bowen, & Faubert, 2020). LCA identifies underlying and unobservable categorical latent variables and thus establishes probabilistic subgroups of LTCs (Kongsted & Nielsen, 2017), which contributes to less biased estimation of class-specific means (Karnowski, 2017). Therefore, it is recommended to categorise a population into a limited number of subgroups with similar LTC combinations for complex issues like multimorbidity (Park, Lee, & Park, 2019). The 35 LTCs were used as observed indicators. We performed several iterations of LCA using different number of classes (three to eight). Akaike information criteria (AIC) and Bayesian information criteria (BIC) were used to inform the optimal number of classes. Models with smaller AIC and BIC are preferable (Olaya et al., 2017).

Upon the analysis and presentation of results, the clinical team including a general practice physician, psychiatrist, and physiotherapist (all with extensive experience in SMI and MLTCs), as well as Patient and Public Involvement (PPI) group of five patients with lived experience of mental and physical health problems, were consulted to determine the most suitable models. After presenting the models and considering different clusters, the team agreed on the optimal number of classes based on AIC, PPI and clinical health expert opinion and each patient with SMI was assigned to a class for which they had the largest posterior probability (i.e. the class they most belonged to). Despite the 3-class model being presented as an alternative option based on BIC, the primary results presented in the current paper are for the 5-class model based on AIC. Additional results for the 3-class model are presented in the Online supplementary material Table S2. After selecting the best model, LTCs were assigned to one class according to a high computed probability of membership (probability >0.3), and each participant was assigned to one class only. The characteristics of patients with SMI in the different latent classes were compared using ANOVA for continuous variables and a chi-square test for categorical variables.

Following the LCA, multinominal logistic regression was conducted to explore associations of sociodemographic and clinical characteristics (i.e. age, gender, ethnicity, and deprivation score) and SMI diagnoses with latent class membership, where the MLTC group was the dependent variable. Associations were quantified using odds ratios (ORs) with 95% confidence intervals (CIs). The largest class membership was selected as the reference group for analyses. All analyses were conducted using STATA 13 software (Stata Corp LP, s.d.).

## Results

The sample included 1924 people with SMI and at least two LTCs with a mean age of 48.2 years (s.d. = 17.3), 49.7% of whom were female. The majority of our sample was from the White (33.8%) or Black (22.7%) ethnic group and had a diagnosis of schizophrenia spectrum disorder (i.e. ICD-10 codes F20*-25*; 56.4%). The distribution of sociodemographic and clinical characteristics in the overall sample and by multimorbidity classes is in Table 1. For our SMI sample, the top three most prevalent LTCs include depression and/or anxiety, chronic pain and psoriasis and/or eczema. Multimorbidity classes displayed significant heterogeneity from the whole population in all characteristics apart from neighbourhood deprivation score.

**Table 1.** Characteristics of total sample and patients with SMI assigned to the 5 multimorbidity classes

	Total sample (*n* = 1924)	Class 1 (*n* = 479)	Class 2 (*n* = 466)	Class 3 (*n* = 585)	Class 4 (*n* = 271)	Class 5 (*n* = 123)	*p* Value^a^
Age (mean, s.d.)	48.2 (17.3)	43.6 (11.6)	39.1 (13.0)	45.3 (13.5)	64.0 (16.3)	78.9 (13.1)	**<0.0001**
Gender (*n*, %)							**<0.0001**
Female	957 (49.7%)	140 (29.2%)	242 (51.9%)	385 (65.8%)	122 (45.0%)	68 (55.3%)	
Male	967 (50.3%)	339 (70.8%)	224 (48.1%)	200 (34.2%)	149 (55.0%)	55 (44.7%)	
Ethnicity^b^ (*n*, %)							**<0.0001**
White	651 (33.8%)	214 (44.7%)	145 (31.1%)	161 (27.5%)	80 (29.5%)	51 (41.5%)	
Black	437 (22.7%)	93 (19.4%)	101 (21.7%)	130 (22.2%)	77 (28.4%)	36 (29.3%)	
Asian	75 (3.9%)	13 (2.7%)	15 (3.2%)	21 (3.6%)	15 (5.5%)	11 (8.9%)	
Mixed/other	225 (11.7%)	48 (10.0%)	48 (10.3%)	90 (15.4%)	32 (11.8%)	7 (5.7%)	
SMI diagnosis (*n*, %)							**<0.0001**
Schizophrenia spectrum disorders	1085 (56.4%)	311 (64.9%)	241 (51.7%)	260 (44.4%)	182 (67.2%)	91 (74.0%)	
Bipolar disorders	517 (26.9%)	115 (24.0%)	160 (34.3%)	190 (32.5%)	44 (16.2%)	8 (6.5%)	
Depressive and other	322 (16.7%)	53 (11.1%)	65 (14.0%)	135 (23.1%)	45 (16.6%)	24 (19.5%)	
Deprivation score (mean, s.d.)	244.6 (116.2)	245.0 (115.0)	243.0 (123.5)	252.6 (113.6)	236.7 (116.2)	228.5 (103.0)	0.168
Long-term conditions (*n*, %)							
Depression/Anxiety	1549 (80.5%)	398 (83.1%)	357 (76.6%)	585 (100%)	129 (47.6%)	80 (65.0%)	**<0.0001**
Chronic pain	1111 (57.7%)	214 (44.7%)	129 (27.7%)	487 (83.3%)	172 (63.5%)	109 (88.6%)	**<0.0001**
Psoriasis/Eczema	624 (32.4%)	101 (21.1%)	313 (67.1%)	123 (21.0%)	43 (15.9%)	44 (35.8%)	**<0.0001**
Asthma	471 (24.5%)	96 (20.0%)	236 (50.6%)	94 (16.1%)	5 (1.9%)	40 (32.5%)	**<0.0001**
Substance dependency	366 (19.0%)	318 (66.4%)	34 (7.3%)	0	7 (2.6%)	7 (5.7%)	**<0.0001**
Hypertension	325 (16.9%)	19 (4.0%)	0	52 (8.9%)	162 (59.8%)	92 (74.8%)	**<0.0001**
Alcohol problem	273 (14.2%)	235 (49.1%)	0	10 (1.7%)	20 (7.4%)	8 (6.5%)	**<0.0001**
Diabetes	238 (12.4%)	12 (2.5%)	2 (0.4%)	59 (10.1%)	102 (37.6%)	63 (51.2%)	**<0.0001**
Hearing problem	208 (10.8%)	37 (7.7%)	73 (15.7%)	18 (3.1%)	23 (8.5%)	57 (46.3%)	**<0.0001**
IBS	169 (8.8%)	29 (6.1%)	13 (2.8%)	109 (18.6%)	1 (0.4%)	17 (13.8%)	**<0.0001**
Blindness/Low vision	115 (6.0%)	8 (1.7%)	7 (1.5%)	0	25 (9.2%)	75 (61.0%)	**<0.0001**
Thyroid disorders	108 (5.6%)	9 (1.9%)	25 (5.4%)	39 (6.7%)	17 (6.3%)	18 (14.6%)	**<0.0001**
HIV/AIDS	98 (5.1%)	70 (14.6%)	12 (2.6%)	10 (1.7%)	5 (1.9%)	1 (0.8%)	**<0.0001**
Chronic kidney disease	88 (4.6%)	1 (0.2%)	0	4 (0.7%)	30 (11.1%)	53 (43.1%)	**<0.0001**
Cancer	82 (4.3%)	8 (1.7%)	21 (4.5%)	5 (0.9%)	25 (9.2%)	23 (18.7%)	**<0.0001**
Epilepsy	75 (3.9%)	18 (3.8%)	29 (6.2%)	0	24 (8.9%)	4 (3.3%)	**<0.0001**
Stroke	74 (3.9%)	1 (0.2%)	1 (0.2%)	0	47 (17.3%)	25 (20.3%)	**<0.0001**
Dementia	58 (3.0%)	0	0	0	23 (8.5%)	35 (28.5%)	**<0.0001**
Chronic liver disease	57 (3.0%)	37 (7.7%)	2 (0.4%)	2 (0.3%)	15 (5.5%)	1 (0.8%)	**<0.0001**
Atrial fibrillation	49 (2.6%)	1 (0.2%)	0	1 (0.2%)	16 (5.9%)	31 (25.2%)	**<0.0001**
Chronic sinusitis	46 (2.4%)	10 (2.1%)	10 (2.2%)	20 (3.4%)	1 (0.4%)	5 (4.1%)	0.057
Coronary heart disease	43 (2.2%)	5 (1.0%)	0	0	19 (7.0%)	19 (15.5%)	**<0.0001**
COPD	42 (2.2%)	12 (2.5%)	0	3 (0.5%)	3 (1.1%)	24 (19.5%)	**<0.0001**
Diverticular disease	40 (2.1%)	4 (0.8%)	0	6 (1.0%)	10 (3.7%)	20 (16.3%)	**<0.0001**
Heart failure	39 (2.0%)	1 (0.1%)	0	6 (1.0%)	10 (3.7%)	22 (17.9%)	**<0.0001**
Prostate disorders	39 (2.0%)	9 (1.9%)	3 (0.6%)	0	15 (5.5%)	12 (9.8%)	**<0.0001**
Anorexia/Bulimia	37 (1.9%)	10 (2.1%)	6 (1.3%)	20 (3.4%)	0	1 (0.8%)	**0.007**
RH/Arthritis	32 (1.7%)	7 (1.5%)	0	15 (2.6%)	3 (1.1%)	7 (5.7%)	**<0.0001**
Learning disabilities	29 (1.5%)	5 (1.0%)	21 (4.5%)	0	0	3 (2.4%)	**<0.0001**
Peptic ulcer	24 (1.3%)	7 (1.5%)	2 (0.4%)	3 (0.5%)	2 (0.7%)	10 (8.1%)	**<0.0001**
Peripheral vascular disease	24 (1.3%)	0	0	0	9 (3.3%)	15 (12.2%)	**<0.0001**
Inflammatory bowel disease	19 (1.0%)	0	6 (1.3%)	11 (1.9%)	1 (0.4%)	1 (0.8%)	**0.026**
Parkinson's disease	10 (0.5%)	0	2 (0.4%)	0	7 (2.6%)	1 (0.8%)	**<0.0001**
MS	9 (0.5%)	0	5 (1.1%)	1 (0.2%)	1 (0.4%)	2 (1.6%)	**0.031**
Bronchiectasis	7 (0.4%)	3 (0.6%)	0	1 (0.2%)	0	3 (2.4%)	**0.001**

IBS, irritable bowel syndrome; COPD, chronic obstructive pulmonary disease; RH, rheumatoid arteritis; MS, multiple sclerosis.

a ANOVA for continuous variables and χ^2^ tests for categorical variables were performed to examine characteristic differences between 5 latent classes; *p* value < 0.05 are marked in bold.

b Variable with missing values.

The number classification of the LCA analysis is shown in Supplementary material Table 1. The AIC values suggested a 5-class model was most suitable, while BIC values suggested a 3-class model. Following expert clinical panel review and PPI group discussion, the 5-class model was judged as the most clinically relevant and was chosen as the optimal classification for this research. The 3- class model is presented in the supplementary material Table 2.

### Subgroups of patients with SMI

**Fig. 1.**
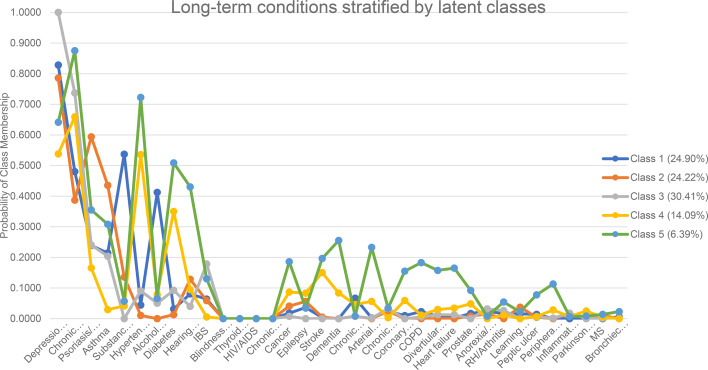
Long-term conditions stratified by 5 latent classes. Class 1: Dependency cluster. Class 2: Atopic cluster. Class 3: Pure affective cluster. Class 4: Cardiovascular cluster. Class 5: Complex multimorbidity cluster. IBS, irritable bowel syndrome; COPD, chronic obstructive pulmonary disease; RH, rheumatoid arteritis; MS, multiple sclerosis.

Multimorbidity classes among patients with SMI are shown in Table 2. All classes presented excess probability of depression/anxiety and chronic pain. Figure 1 shows the probability of each LTC in all 5 classes. On average, the number of LTC patients in each cluster has ranged from 7 to 9.

**Table 2.** Predicted probabilities of latent multimorbidity 5-class membership

	Class 1 Dependency cluster	Class 2 Atopic cluster	Class 3 Pure affective cluster	Class 4 Cardiovascular cluster	Class 5 Complex multimorbidity cluster
Probability (class)	0.27	0.25	0.26	0.15	0.07
Probability of					
Depression/anxiety	**0.83**	**0.79**	**1.00**	**0.54**	**0.64**
Chronic pain	**0.48**	**0.39**	**0.74**	**0.66**	**0.88**
Psoriasis/Eczema	0.24	**0.59**	0.24	0.17	**0.36**
Asthma	0.21	**0.44**	0.20	0.03	**0.31**
Substance dependency	**0.54**	0.14	<0.01	0.04	0.06
Hypertension	0.04	0.01	0.09	**0.54**	**0.72**
Alcohol problem	**0.41**	<0.01	0.05	0.08	0.07
Diabetes	0.03	0.01	0.09	**0.35**	**0.51**
Hearing problem	0.08	0.13	0.04	0.09	**0.43**
IBS	0.06	0.06	0.18	0.01	0.13
Blindness/Low vision	0.02	0.02	<0.01	0.09	**0.54**
Thyroid disorders	0.02	0.05	0.06	0.07	0.15
HIV/AIDS	0.12	0.03	0.02	0.02	0.01
Chronic kidney disease	<0.01	<0.01	0.01	0.11	**0.39**
Cancer	0.02	0.04	0.01	0.09	0.19
Epilepsy	0.04	0.06	<0.01	0.08	0.03
Stroke	<0.01	<0.01	<0.01	0.15	0.20
Dementia	<0.01	<0.01	<0.01	0.08	0.26
Chronic liver disease	0.07	0.01	0.01	0.05	0.01
Atrial fibrillation	<0.01	<0.01	<0.01	0.06	0.23
Chronic sinusitis	0.02	0.02	0.03	<0.01	0.03
Coronary heart disease	0.01	<0.01	<0.01	0.06	0.16
COPD	0.02	<0.01	0.01	0.01	0.18
Diverticular disease	0.01	<0.01	0.01	0.03	0.16
Heart failure	<0.01	<0.01	0.01	0.03	0.16
Prostate disorders	0.02	0.01	<0.01	0.05	0.09
Anorexia/Bulimia	0.02	0.02	0.03	<0.01	0.01
RH/Arthritis	0.02	<0.01	0.03	0.01	0.05
Learning disability	0.02	0.04	<0.01	<0.01	0.02
Peptic ulcer	0.01	<0.01	0.01	0.01	0.08
Peripheral vascular disease	<0.01	<0.01	<0.01	0.03	0.11
Inflammatory bowel disease	<0.01	0.02	0.02	0.01	0.01
Parkinson's disease	<0.01	<0.01	<0.01	0.02	0.01
MS	<0.01	0.01	<0.01	<0.01	0.01
Bronchiectasis	0.01	<0.01	<0.01	<0.01	0.02
Proportion of participants in each class	24.9%	24.2%	30.4%	14.0%	6.4%
Mean number of LTCs^a^	7	7	7	8	9

IBS, irritable bowel syndrome; COPD, chronic obstructive pulmonary disease; RH, rheumatoid arteritis; MS, multiple sclerosis; LTCs, long-term conditions.

LTCs with a high probability (>0.3) are marked in bold.

a Mean number of 35 included LTCs (excluding SMI).

Class 1, the ‘substance-related cluster’, included over 20% patients with SMI (*n* = 479; 24.9%). This class presented a high probability of substance dependency (probability = 0.54) and alcohol problem (0.41).

Class 2, the ‘atopic cluster’, consisted of a similar percentage of patients with SMI (*n* = 466, 24.2%). This class presented a high probability of psoriasis/eczema (0.59) and asthma (0.44).

Class 3, the ‘pure affective cluster’, contained approximately one third of the overall sample (*n* = 585, 30.4%). This class had a high probability of depression/anxiety (1.00) and chronic pain (0.74) with relatively lower probability for other LTCs, compared to other clusters.

Class 4, the ‘cardiovascular cluster’, was assigned to 14.1% (*n* = 271) of the sample. This class showed a high probability of hypertension (0.54) and diabetes (0.35).

Class 5, ‘complex multimorbidity cluster’, included about 6.4% (*n* = 123) of the sample. This class had a relatively high probability of a number of LTCs, including psoriasis/eczema (0.36), asthma (0.31), hypertension (0.72), diabetes (0.51), hearing problem (0.43), blindness/low vision (0.54) and chronic kidney disease (0.39).

### Multinominal logistic regression analysis

The results of the multinominal logistic regression analysis are shown in Table 3. Compared to the pure affective cluster (i.e. the largest cluster and thus applied as the reference cluster), the substance-related cluster tended to be younger, male, more likely to be from a White ethnic background, and more likely to have a diagnosis of a schizophrenia spectrum disorder (than bipolar disorders, depressive disorders and other). Similarly, the atopic cluster also tended to be younger and more male than the reference cluster. Compared to the reference cluster, the cardiovascular cluster was older, male, more likely to be from a Black ethnic background, and more likely to have a diagnosis of schizophrenia spectrum disorder. Additionally, compared to the reference cluster, the complex multimorbidity cluster appeared to be the oldest among all clusters (i.e. with the highest OR for age) and more likely to be male.

**Table 3.** Multinominal logistic regression between latent classes and covariates, with class 3 as the reference class

Variables	Latent classes												
	Class 1 Dependency cluster			Class 2 Atopic cluster			Class 4 Cardiovascular cluster			Class 5 Complex multimorbidity cluster			
	OR	95% CI	*p* Value	OR	95% CI	*p* Value	OR	95% CI	*p* Value	OR	95% CI	*p* Value	
Age (One-year increase)	0.99	0.98–1.00	**0.004**	0.96	0.95–0.97	**<0.0001**	1.09	1.07–1.10	**<0.0001**	1.16	1.14–1.18	**<0.0001**	
Gender													
Female	Reference			Reference			Reference			Reference			
Male	4.28	3.27–5.60	**<0.0001**	1.75	1.35–2.28	**<0.0001**	2.82	2.01–3.94	**<0.0001**	2.71	1.63–4.51		**<0.0001**
Ethnicity													
White	Reference			Reference			Reference			Reference			
Black	0.46	0.32–0.66	**<0.0001**	0.72	0.50–1.04	0.084	1.75	1.10–2.79	**0.018**	1.55	0.81–2.95		0.187
Asian	0.39	0.18–0.84	**0.016**	0.71	0.34–1.48	0.364	1.27	0.55–2.92	0.575	1.46	0.51–4.13		0.480
Mixed/other	0.41	0.27–0.63	**<0.0001**	0.53	0.35–0.82	**0.004**	1.47	0.85–2.54	0.172	1.05	0.39–2.79		0.928
SMI diagnosis													
Schizophrenia spectrum disorders^a^	Reference			Reference			Reference			Reference			
Bipolar disorders^b^	0.62	0.45–0.84	**0.003**	0.86	0.63–1.16	0.313	0.57	0.37–0.87	**0.010**	0.27	0.11–0.64		**0.003**
Depressive and other	0.40	0.27–0.58	**<0.0001**	0.60	0.42–0.85	**0.005**	0.50	0.32–0.77	**0.002**	0.57	0.30–1.60		0.076
Deprivation score	1.00	0.999–1.00	0.63	1.00	0.998–1.00	0.053	1.00	0.998–1.00	0.507	1.00	0.998–1.00		0.545

a ICD-10 F20*-25, F28*-29*.

b ICD-10 F30*-31*.

*p* value <0.05 are marked in bold.

## Discussion

To the best of our knowledge, this paper is the first to examine the epidemiology of data-driven latent classes of more complex multimorbidity as well as associated risk factors among patients with SMI and complex needs (i.e. at least two existing LTCs). This study identified 5 latent classes: ‘substance related’, ‘atopic’, ‘pure affective’, ‘cardiovascular’, and ‘complex multimorbidity’ clusters. Against the ‘pure affective’ cluster, age, gender, ethnic background and SMI diagnoses were identified as significant factors for the other clusters. This study has important implications, suggesting the presentation of MLTCs is common and complex in people with SMI. The study also advances knowledge in understanding complex multimorbidity including 5 MLTCs and found that people with SMI on average had 7–9 LTCs in each class. Previous studies have only focussed on SMI and fewer MLTCs and neglected the fact complexity of this relationship of complex clusters among people with SMI (Launders, Hayes, Price, & Osborn, 2021; Stubbs et al., 2016).

The potential causes of this high prevalence of LTCs and multimorbidity in this population are multifaceted. A number of risk factors, like lifestyle factors, health behaviours, and medications (De Hert et al., 2011), may increase the risk of developing LTCs and multimorbidity in this population. It has been established that a mental health diagnosis itself is a risk factor for LTCs and multimorbidity (Lindqvist et al., 2015); this is further compounded by healthcare inequalities experienced by this population. Published evidence has suggested poor screening and treatment rate for physical comorbidities among patients with SMI (Firth et al., 2019), including cardiovascular disease (Solmi et al., 2021) and cancer (Solmi et al., 2020), as well as a lack of integrated care in primary and secondary healthcare services, with individual physical conditions being more widely treated than multimorbidity (Sinnige et al., 2013). Furthermore, interpersonal stigma (Perese & Wolf, 2005), loneliness (Lauder, Sharkey, & Mummery, 2004) and poor social support (Munikanan et al., 2017) could lead to prolonged stress and further compromise patients' immune system, increase the risk of inflammation (Goodell, Druss, Walker, & Mat, 2011), thus increase their vulnerability to chronic illnesses.

Finding that some included LTCs had a low prevalence among our sample is surprising, such as chronic liver disease, stroke and cancer, given the high possibility of obesity, smoking, alcohol misuse, and potential side effects of medications in this population (De Hert et al., 2011). This might be related to known under-recording of physical LTCs in SMI patients and to how physical conditions are more likely to be recorded at the time of death for people with schizophrenia (Heiberg et al., 2019). Of the LTCs included in our study, 17 feature in the Quality and Outcomes Framework [QOF]. Since only QOF-specified conditions are incentivised, bias is expected, and incentivised illnesses are more likely to be screened and recorded. Moreover, diagnostic overshadowing and underdiagnosis is common in those with SMI (Firth et al., 2019; Solmi et al., 2021). Additionally, the current study included a relatively young sample (i.e. mean age of 48.2 years), which may further explain a low prevalence of certain diseases identified.

The presence of depression and/or anxiety across all clusters is not surprising. Both anxiety and depression are common in LTCs (Firth et al., 2019), with a prevalence of depressive disorders reaching 80% in people with schizophrenia (Upthegrove, Marwaha, & Birchwood, 2017). Anxiety symptoms and related disorders could also occur in up to 38% of patients with schizophrenia (Temmingh & Stein, 2015). Our results further underscore the importance of affective disorders as a comorbidity of all different SMI typologies.

The high prevalence of chronic pain in our sample is unexpected (i.e. 57.7% in total). However, there is an increasing recognition that chronic pain and mental illness tends to co-occur, with approximately 33% of patients with SMI (Stubbs et al., 2014) experiencing chronic pain. High rates of chronic pain have also been reported by patients with major depressive disorders and bipolar disorders (Owen-Smith et al., 2020; Stubbs et al., 2015a, 2015b, 2015c). However, chronic pain has received little attention in people with SMI, despite reduced pain sensitivity (Stubbs et al., 2015a, 2015b, 2015c) and higher severity of pain (Strassnig, Brar, & Ganguli, 2003) reported by people with schizophrenia. Growing evidence also suggests an underreporting of pain (Kuritzky, Mazeh, & Levi, 1999) and low treatment rate for pain (de Almeida, Braga, Lotufo Neto, & de mattos Pimenta, 2013; Stubbs et al., 2015b) among people with schizophrenia. Despite the high prevalence of pain (Stubbs et al., 2014), potential under reporting/treatment and the association with low quality of life (Stubbs et al., 2015b), research and clinical guidelines on pain in SMI are largely absent. Given the high prevalence of chronic pain recorded, the current study emphasises an urgent need for increased awareness, identification, and treatment of chronic pain in patients with SMI.

Regarding the substance-related cluster, substance-related disorders are commonly experienced by people with SMI (Kavanagh, McGrath, Saunders, Dore, & Clark, 2002), with approximately 50% experiencing substance use disorder (Kendler, 1996). More specifically, for people with schizophrenia, the lifetime prevalence can reach 86% for alcohol, 83% for cannabis (Volkow, 2009) and 50% for cocaine use (Chambers, Krystal, & Self, 2001). The co-occurrence of substance-related disorders and SMI has serious consequences, including treatment nonadherence, increased risk for other illnesses such as HIV (Brunette & Mueser, 2006), relapse, suicidality and re-hospitalisation (van Dijk, Koeter, Hijman, Kahn, & van den Brink, 2012). Our study also demonstrates a high risk of substance-related disorders in younger White males with SMI, in line with previous research (Dixon, 1999; Foti, Kotov, Guey, & Bromet, 2010; Graham et al., 2001; Schofield, Quinn, Haddock, & Barrowclough, 2001). A diagnosis of schizophrenia and schizotypal spectrum clusters appears to be another risk factor for dual diagnosis (Graham et al., 2001).

The co-occurrence of asthma and psoriasis/eczema are high in the atopic cluster. The association between asthma and psoriasis has been previously confirmed in the general population (Fang, Liao, Lin, Chen, & Kao, 2015; Wang et al., 2018), possibly due to long-term inflammation in the airway (Wang et al., 2018) and overlapping genetic risk (Weidinger et al., 2013). Psoriasis (Ferreira, Abreu, Reis, & Figueiredo, 2016) and asthma (Wu et al., 2019) have been associated with a range of psychiatric disorders, including bipolar disorder, psychotic and neurotic spectrum disorders. However, their relationship and compresence have been less explored in people with SMI. A longitudinal Danish study confirmed an association between atopic disorders and an increased risk of schizophrenia (Pedersen, Benros, Agerbo, Børglum, & Mortensen, 2012). Another large-scaled case-control study also established a relationship between psoriasis and schizophrenia spectrum study using a population-wide database (Carvalho et al., 2021). Therefore, the current study provides further support to these findings and demonstrates a particular at-risk group (i.e. younger male) who may experience both SMI, asthma and psoriasis/eczema.

The cardiovascular cluster consists of a high prevalence of hypertension and diabetes. Hypertension is a common risk factor for cardiovascular diseases, and is particularly common in diabetes (Cryer, Horani, & DiPette, 2016). People with SMI are at 2–3 odds more likely to have metabolic syndrome, diabetes or cardiovascular disease compared to the general population (Correll et al., 2017; Vancampfort et al., 2016, 2015). Therefore, this cluster is not surprising and is where the bulk of epidemiology and intervention research has focussed (Firth et al., 2019).

The current study also identified a complex multimorbidity cluster, which consists of a wide range of LTCs. Patients in this cluster are also identified as older and male, which might partially explain the high number of LTCs present in this cluster, as the number of chronic conditions tend to increase with age (Ofori-Asenso et al., 2019; Ryan et al., 2018). Ethnicity was not found to be associated with this cluster, so is maybe less of a factor in predicting health inequalities in older people in general.

This study provides novel evidence on the prevalence of LTCs, patterns and prevalence of multimorbidity latent classes among patients with SMI and complex needs. Despite a recent shift in improving the physical health of people with SMI (Firth et al., 2019), research has just started to consider MLTCs in SMI. There is a need for an integrated care approach for patients with SMI (Firth et al., 2019), involving healthcare professionals which may have a long-term benefit in treating multimorbidity (Firth et al., 2019; Smith, Soubhi, Fortin, Hudon, & O'Dowd, 2012). Just like incentivisation for the screening of QOF indicators, joint multimorbidity work may also be incentivised to encourage better management of multimorbidity and efficient communication between psychiatrists and physicians.

The current paper identifies 5 clusters of patients with SMI who may be at risk of specific patterns of multimorbidity, highlighting a need for prevention. Healthcare providers should pay attention to people with specific multimorbidity patterns, and new interventions should be developed accordingly, paying attention to specific sociodemographic characteristics. Physicians can play a vital role in identifying these patients who are at high-risk of complex multimorbidity clusters, referring them to appropriate interventions based on their individual needs and clinical complexity (Powers & Chaguturu, 2016).

This study benefits from a range of strengths, like the use of a large, up-to-date representative population-based sample and a comprehensive list of LTCs derived from primary healthcare records. Furthermore, this study includes both included patients with SMI in primary care data and patients who were known to secondary mental health services, offering accuracy and representativity and potentially including individuals on a more severe/complex end of the SMI spectrum. While we explored latent classes of more complex multimorbidity, findings should be interpreted considering the following limitations. First, our study is restricted to one geographic area, the borough of Lambeth in South East London, which is characterised by a multi-ethnic, urban population with high levels of deprivation. Therefore, our results may not extend to other areas in London or the whole UK population. Furthermore, our sample comprised of a relatively young population, and this may explain a low prevalence of certain LTCs found in our sample, such as cancer and stroke. Our results, therefore, may not be generalised to an older population. Second, health administrative data do not consider other potential covariates and can be inconsistently recorded. Third, we did not match our sample to controls to compare the multimorbidity clusters. However, previous research has indicated similar multimorbidity clusters between the two populations (Filipčić et al., 2018; Launders et al., 2021). Fourth, the terms for clusters in this study are descriptive and only give a ‘snapshot’ of the clusters, making our descriptions imperfect. Additionally, following an expert clinical panel review and a discussion within the PPI group, we decided on a 5-class model instead of a 3-class model, and some may consider our clustering is arbitrary. However, the decision was made with a full consideration of health professionals' clinical opinions and driven by real life experiences from patients with lived experience of both mental and physical illnesses. Fifth, given the current study only included patients with SMI who had a record on both LDN and CRIS, patients who were not in contact with primary and secondary healthcare services may have been omitted. Sixth, due to the nature of the study, the directional association of SMI and LTCs cannot be established. Sixth, given this is the first study examining multimorbidity clusters in patients with SMI and complex needs, clusters generated in the current research could not be compared to existing research. Future research from independent groups should seek to replicate our findings. Lastly, we did not investigate the severity of LTCs in people with SMI, and we did not retrieve additional data on clinical outcomes such as GP consultation rate, hence, future studies may wish to explore these outcomes.

## Conclusion

The current study is, to the best of our knowledge, one of the first to investigate multimorbidity clusters among people with SMI and two or more existing LTCs. We identified a high prevalence of at least seven MLTCs across five clusters within our sample and explored potential risk factors that may contribute to these clusters. Given the high prevalence of LTCs and complex multimorbidity clusters, as well as health inequalities experienced by people with SMI, an integrated care approach for treating multimorbidity and a tailored approach to manage and treat patients in the identified five clusters identified are recommended.

## Supplementary material

For supplementary material accompanying this paper visit https://doi.org/10.1017/S003329172200109X. click here to view supplementary material
