# Supplementary material for: Multimorbidity clusters among people with serious mental illness: a representative primary and secondary data linkage cohort study
Source: Psychol Med. 2022 Apr 29;53(10):4333–44. doi: 10.1017/S003329172200109X (PMC10388332; doi:10.1017/S003329172200109X)
Supplement: Supplementary file 1 [file S003329172200109Xsup001.docx]

**Supplementary material**

| **Table 1.** Akaike Information Criteria (AIC) and Bayesian Information Criteria (BIC) for each potential model. | | |
| --- | --- | --- |
|  | *AIC* | *BIC* |
| 8-class model | 27617.016 | 29207.794 |
| 7-class model | 27590.601 | 28986.703 |
| 6-class model | 27596.271 | 28792.136 |
| 5-class model | 27647.549 | 28643.176 |
| 4-class model | 27695.107 | 28484.934 |
| 3-class model | 27820.865 | 28416.016 |

**Table 2**. Predicted probabilities of latent multimorbidity 3-class membership

|  | **Class 1** | **Class 2** | **Class 3** |  |  |
| --- | --- | --- | --- | --- | --- |
| Probability (class) | 0.34 | 0.47 | 0.19 |  |  |
| *Probability of* |  |  |  |  |  |
| *Depression/anxiety* | **0.81** | **0.89** | **0.57** |  |  |
| *Chronic pain* | **0.48** | **0.57** | **0.76** |  |  |
| *Psoriasis/Eczema* | 0.28 | **0.39** | 0.23 |  |  |
| *Asthma* | 0.24 | 0.29 | 0.15 |  |  |
| *Substance dependency* | **0.51** | 0.02 | 0.04 |  |  |
| *Hypertension* | 0.05 | 0.07 | **0.62** |  |  |
| *Alcohol problem* | **0.35** | 0.02 | 0.07 |  |  |
| *Diabetes* | 0.04 | 0.07 | **0.43** |  |  |
| *Hearing problem* | 0.08 | 0.08 | 0.23 |  |  |
| *IBS* | 0.06 | 0.12 | 0.06 |  |  |
| *Blindness/Low vision* | 0.02 | 0.01 | 0.27 |  |  |
| *Thyroid disorders* | 0.02 | 0.06 | 0.10 |  |  |
| *HIV/AIDS* | 0.11 | 0.03 | 0.01 |  |  |
| *Chronic kidney disease* | <0.01 | <0.01 | 0.24 |  |  |
| *Cancer* | 0.02 | 0.03 | 0.13 |  |  |
| *Epilepsy* | 0.04 | 0.03 | 0.06 |  |  |
| *Stroke* | 0.01 | 0.01 | 0.18 |  |  |
| *Dementia* | <0.01 | <0.01 | 0.16 |  |  |
| *Chronic liver disease* | 0.06 | 0.01 | 0.03 |  |  |
| *Atrial fibrillation* | <0.01 | <0.01 | 0.13 |  |  |
| *Chronic sinusitis* | 0.02 | 0.03 | 0.01 |  |  |
| *Coronary heart disease* | 0.01 | <0.01 | 0.10 |  |  |
| *COPD* | 0.02 | <0.01 | 0.08 |  |  |
| *Diverticular disease* | 0.01 | <0.01 | 0.09 |  |  |
| *Heart failure* | <0.01 | <0.01 | 0.09 |  |  |
| *Prostate disorders* | 0.02 | 0.01 | 0.07 |  |  |
| *Anorexia/Bulimia* | 0.02 | 0.03 | <0.01 |  |  |
| *RH/Arthritis* | 0.01 | 0.01 | 0.03 |  |  |
| *Learning disability* | 0.02 | 0.01 | 0.01 |  |  |
| *Peptic ulcer* | 0.01 | <0.01 | 0.04 |  |  |
| *Peripheral vascular*  *disease* | <0.01 | <0.01 | 0.06 |  |  |
| *Inflammatory bowel*  *disease* | <0.01 | 0.02 | <0.01 |  |  |
| *Parkinson’s disease* | <0.01 | <0.01 | 0.02 |  |  |
| *MS* | <0.01 | <0.01 | 0.01 |  |  |
| *Bronchiectasis* | <0.01 | <0.01 | 0.01 |  |  |
| Proportion of participants in each class | 28.17% | 53.85% | 17.98% |  |  |
|  |  |  |  |  |  |

Abbreviations: IBS (irritable bowel syndrome); COPD (chronic obstructive pulmonary disease); RH (rheumatoid arteritis); MS (multiple sclerosis); LTCs (long-term conditions)

LTCs with a high probability (>0.3) are marked in bold
